# The Association Between Nurse Managers’ Trust, Inclusive Leadership Style and Staff Nurses’ Work Effectiveness in Saudi Arabian Hospitals: A Descriptive Cross-Sectional Study

**DOI:** 10.3390/healthcare14152353

**Published:** 2026-08-02

**Authors:** Reem AL-Dossary, Noura Almadani, Maram Banakhar, Hibah Bahri

**Affiliations:** 1 Nursing Education Department, College of Nursing, Imam Abdulrahman Bin Faisal University, Dammam 31441, Saudi Arabia; 2 Community and Psychiatric Mental Health Nursing Department, College of Nursing, Princess Nourah Bint Abdulrahman University, Riyadh 11432, Saudi Arabia; naalmadani@pnu.edu.sa; 3 Public Health Department, Faculty of Nursing, King Abdulaziz University, Jeddah 21589, Saudi Arabia; ahbbanakher3@kau.edu.sa; 4 Medical-Surgical Department, College of Nursing, Princess Nourah Bint Abdulrahman University, Riyadh 11432, Saudi Arabia; habahri@pnu.edu.sa

**Keywords:** trust, nursing managers, inclusive leadership, nurses, work effectiveness, hospitals

## Abstract

**Background:** Nurse managers’ trust and inclusive leadership are critical factors that influence staff nurses’ motivation, engagement, and work effectiveness. Understanding how these elements interact can help improve organizational performance and patient care in hospital settings. Therefore, this study aimed to examine the relationship between nursing managers’ trust, inclusive leadership styles and staff nurses’ work effectiveness in Saudi Arabian hospitals. **Purpose:** This study aimed to examine the relationship between nursing managers’ trust, inclusive leadership styles and staff nurses’ work effectiveness in Saudi Arabian hospitals. **Methods:** A descriptive, correlational cross-sectional design was conducted. Data were collected by distributing questionnaires (workplace effectiveness questionnaire II, the inclusive leadership scale, and the trust in the leader scale) among 487 nurses using convenience sampling from 10 hospitals in Saudi Arabia. **Results:** The work-effectiveness factors of opportunity, access to support, and access to information (means: 3.43, 3.23, and 3.15, respectively) were moderately empowering. Most participants perceived the availability and accessibility (means: 3.43 and 3.23, respectively) of inclusive leadership. Meanwhile, in the trust in the leader scale, integrity and benevolence (means: 3.31 and 3.27, respectively) were more highly rated than predictability and competence (means: 2.79 and 2.81, respectively). Significant differences (*p* < 0.05) between nursing managers and their staff were observed across all three dimensions. **Conclusions:** Trust in nursing managers, inclusive leadership, and staff nurses’ work effectiveness were significantly associated, but the cross-sectional design does not support causal inference.

## 1. Introduction

Trust is an essential component of any successful professional relationship, particularly between nursing managers and their staff. In healthcare organizations, this form of trust supports a collaborative work environment, where nurses feel valued, supported, and empowered to perform their duties effectively [1,2,3]. Trust in leadership refers to the extent to which individuals believe their leaders are competent, reliable, and act with integrity [4]. Studies have shown that this type of trust positively correlates with job satisfaction, organizational commitment, and work performance among employees [5,6], while also reducing turnover, absenteeism, and counterproductive behaviors [7,8]. In the healthcare setting, nurses who trust their managers are more likely to feel secure in their roles and committed to their responsibilities [9]. Nurse managers who actively foster trust can promote job satisfaction, reduce burnout, and improve communication within teams, ultimately contributing to enhanced organizational effectiveness and patient care outcomes [10].

Furthermore, confidence in leadership is a crucial aspect of organizational work effectiveness. When trust is demonstrated among leaders, employees tend to exhibit higher levels of motivation, engagement, and commitment, thereby enhancing productivity and overall performance [10,11,12,13]. Moreover, trust encourages open communication and a willingness to share ideas and take risks without fearing adverse outcomes [11]. This process results in a collaborative, innovative work environment, enhancing the overall effectiveness of the organization. By contrast, a lack of trust can lead to disengagement, decreased motivation, and a lack of commitment to responsibilities, potentially resulting in decreased productivity, subpar performance, and an increased turnover rate [7]. Therefore, employees’ confidence in leadership plays a vital role in establishing a constructive workplace atmosphere. Co-nurses are essential in delivering healthcare services, and their effectiveness is vital for achieving positive patient outcomes [14].

In this regard, an inclusive leadership approach fosters diverse viewpoints and promotes open communication, enhancing collaborations and creativity [15,16]. Therefore, understanding the relationship between the nursing staff’s trust in nursing managers and an inclusive leadership style with respective to the staff’s work effectiveness is crucial for fostering a supportive and thriving healthcare environment. It can provide insights into effective leadership strategies, empower professional growth, and establish a foundation for effective healthcare management.

Inclusive leadership is exemplified by effectively managing and leading a diverse group of individuals while honoring and appreciating each of them for their unique qualities compassionately and without discrimination. This approach rejects discrimination, bias, and favoritism related to color, race, and other characteristics, allowing all employees feel that their contributions are genuinely valued [17,18]. Inclusive leadership is crucial in establishing trust and cultivating a positive workplace atmosphere [18]. By adopting inclusive practices, nursing managers can foster a sense of belonging and enable the staff to embrace risks, innovate, and address challenges effectively. Studies [19,20,21] have determined that factors such as education and training, communication skills, time management, compassion and empathy, and teamwork can influence nurse effectiveness. By focusing on these aspects, nurses can improve their professional performance and provide superior patient care.

Furthermore, inclusive leadership plays a crucial role in fostering trust and has a significant positive effect on work effectiveness [19]. Trust in leadership is characterized as a situation that remains “uncompromised by doubt,” where employees anticipate fair treatment from their leaders, leading to a sense of ease in communicating openly with them [22]. Inclusive leadership also plays a key role in shaping trust. Leaders who consistently model fairness, transparency, and supportiveness are more likely to earn the confidence of their teams [16]. In turn, trust enhances employees’ willingness to commit to organizational goals, engage in continuous learning, and participate in change initiatives [23,24].

In nursing, the presence of inclusive leadership and trust has a direct impact on work effectiveness. Nurses who feel included and supported by their managers are more likely to take initiative, share ideas, and assume greater responsibilities [25,26]. This not only improves their individual performance but also strengthens team dynamics and reduces the workload on managers [27]. In summary, the establishment of trust and the adoption of an inclusive leadership approach are critical elements in fostering a successful relationship between nurse managers and their staff nurses [28], ultimately leading to innovative work behavior [29] and enhanced job performance [30].

According to Alilyyani [31], nursing managers’ inclusive leadership styles is crucial in fostering trust among staff nurses. This approach emphasizes collaboration, fosters open communication, and upholds respect for nursing professionals, facilitating an environment where staff nurses can express their thoughts and feelings freely while being receptive to suggestions and feedback. The objective should be to encourage dialogue and adopt a collaborative strategy that enables collective decision-making. This involves steering clear of discrimination, microaggressions, and language or subjects that could be perceived as offensive [32]. Moreover, nurse managers must be attentive to the needs of their staff and be available for support and guidance. This may involve providing professional development opportunities and guaranteeing that nurses possess the necessary resources to perform their duties effectively.

Moreover, trust-based relationships between nursing managers and staff nurses is crucial in any healthcare environment, especially in Saudi Arabia, which features several cultural nuances and sensitivities within the nursing workforce. An inclusive leadership approach is essential for establishing and sustaining trust in this environment. However, despite the importance of this relationship between trust and inclusive leadership styles, there remains a gap in the literature in relation to the work effectiveness of staff nurses. For instance, a comparative study [33] examined how inclusive leadership affected the psychological distress and job satisfaction of nurses in three Arab nations, including Saudi Arabia, during the COVID-19 pandemic. Although the study revealed strong correlations among job satisfaction, psychological distress, and inclusive leadership, it did not examine the relationship between trust in nurse managers and work effectiveness.

Another study examined trust among nurse managers (supervisors and head nurses) and its relationship with nurses’ performance; however, it did not consider the influence of inclusive leadership [7]. Work effectiveness refers to the means through which an organization, team, or individual can achieve their goals and objectives while making optimal use of available resources. It considers various aspects, including overall performance and productivity, work quality, operational effectiveness, originality, communication, and teamwork [34,35].

The relevance of this inquiry is particularly important in Saudi Arabia, where the nursing workforce remains highly dependent on expatriate nurses. Previous reports [36,37] indicate that approximately 60–70% of nurses in Saudi Arabia are expatriates, and recent national data show that nurse distribution remains uneven across regions, with nurse-to-population ratios ranging from 3.13 to 9.89 per 1000 people. These patterns highlight continuing workforce diversity and structural challenges within Saudi hospitals that may shape nurses’ experiences of leadership and empowerment. In Saudi Arabia, where the nursing workforce is culturally diverse and includes both Saudi and non-Saudi professionals, leadership practices may be shaped by workforce composition, communication norms, and organizational hierarchy. Understanding how nurse leaders and managers influence staff nurses’ perceptions of trust, inclusion, and work effectiveness is therefore essential for informing leadership practices that are responsive to this unique context.

Given the complexity and diversity of healthcare environments in Saudi Arabia, examining this relationship is crucial to firstly improve staff nurses’ performance by understanding the factors that enhance work effectiveness and promote motivation, job satisfaction, and professional growth among staff nurses. Moreover, effective leadership and trust-based relationships create a supportive work environment where nurses can perform at their best. This directly impacts the quality of patient care, safety outcomes, and overall organizational effectiveness. Additionally, while international literature highlights the role of leadership in nursing outcomes, there is limited empirical evidence from the Saudi context. Thus, this study adds to the body of knowledge by addressing cultural and organizational factors unique to Saudi hospitals. This study aims to examine the relationship between nursing managers’ trust, inclusive leadership styles and staff nurses’ work effectiveness in Saudi Arabian hospitals. Accordingly, this study addressed the following research questions:

1. What is the level of trust in nursing managers among staff nurses and nurse managers?
2. What are the perceptions of inclusive leadership and work effectiveness among the participants?
3. Is there a significant relationship between trust in nursing managers, inclusive leadership, and work effectiveness?
4. Do these perceptions differ between nurse managers and staff nurses?

### Theoretical Framework

This study is guided by Social Exchange Theory, which posits that interpersonal relationships in organizations are shaped by reciprocal exchanges of support, trust, and obligation. In nursing contexts, when staff nurses perceive their nurse managers as trustworthy, accessible, and inclusive, they are more likely to reciprocate with stronger engagement, empowerment, and positive perceptions of work effectiveness [38,39]. This framework provides a useful basis for understanding the interrelationships among trust in leaders, inclusive leadership, and work effectiveness.

## 2. Materials and Methods

### 2.1. Study Design and Setting

A descriptive correlational cross-sectional design was conducted. Data on trust in nursing managers and inclusive leadership styles concerning the staff’s job performance were collected from staff nurses in 10 hospitals in Saudi Arabia. These hospitals comprised seven public and three private hospitals.

### 2.2. Study Sample

A convenience sampling approach was employed [40], with two distinct sample groups: nursing managers and staff nurses. The nursing managers provided insights regarding their leadership styles, whereas the staff nurses contributed data regarding their work performance and their trust in their managers. Registered nurses working in the selected hospitals who had at least 1 year of clinical experience were eligible for participation. Nurses who were agency or temporary staff, on sick leave, on leave during the data-collection period, or otherwise not actively working in the participating hospitals were excluded.

Cochran’s formula [41] was used to determine the sample size. The process indicated that from a total of 693 nurses and nurse managers working in the selected hospitals, at a 95% confidence interval and 5% margin of error, a minimum of 248 nurses must be recruited.

### 2.3. Data Collection

The study’s questionnaire included four sections. Section one: *Sociodemographic data*. This instrument collected the nurses’ demographic and work-related data. Section two: Workplace Effectiveness Questionnaire II (CWEQ-II). This tool comprises 19 items designed to assess nursing professionals’ structural empowerment [42,43]. The questionnaire incorporates seven distinct subscales: opportunity (three items), resources (three items), information (three items), support (three items), formal power (three items; job activity scale), informal power (four items; organization relationship scale), and global empowerment (two items). These subscales employ a 5-item Likert scale, with scores ranging from 1 to 5, with higher scores indicating improved access to empowerment structures [38]. Section three: Inclusive leadership. This nine-item tool is designed to capture three major dimensions of inclusive leadership (three items each): openness, availability, and accessibility. The participants were asked to evaluate the degree to which their manager demonstrates openness and is available to them in the workplace on a five-point scale (1: “not at all”; 5: “at all times”) [44]. The inclusive leadership scale was completed by nursing managers as a self-report measure of their own leadership behavior and by staff nurses as an other-report measure reflecting their perceptions of their immediate managers. The same conceptual domains were assessed in both groups, with the referent adjusted to match the respondent perspective. Responses were analyzed at the individual level; no dyadic pairing between specific managers and specific staff nurses was performed unless otherwise stated. Therefore, comparisons between nursing managers and staff nurses represent independent group comparisons rather than matched-pair analyses. Section four: Trust in the leader scale. This 20-item tool aims to assess the four key dimensions of trust, with five items for each dimension. First, competence, or the degree to which an individual demonstrates a set of skills, abilities, and traits that enable them to exert influence in a particular area. Second, integrity, referring to how honorable a person is perceived to be, particularly in aligning their words with their actions. Third, benevolence, reflecting the extent to which an individual is viewed as genuinely caring for others. Finally, predictability, referring to the degree of consistency in an individual’s behavior. The participants were asked to evaluate the degree to which their manager demonstrates openness and is approachable on a five-point scale (1: “strongly disagree”; 5: “strongly agree”) [45].

All three study instruments were made available to participants in both English and Arabic to accommodate the multilingual nursing workforce in Saudi Arabian hospitals. The inclusive leadership scale and the Conditions for Work Effectiveness Questionnaire-II (CWEQ-II) were used in their available validated Arabic versions [46,47] alongside the original English versions. The trust in leader scale, which did not have an established Arabic version suitable for this study population, was translated into Arabic for the present study. The translation process included forward translation, back-translation, and review by bilingual experts to ensure semantic equivalence, conceptual consistency, and cultural appropriateness. The final Arabic wording was reviewed for clarity and suitability before data collection.

Table 1 presents the Cronbach’s alpha values of all subscales (>0.70) of the three questionnaires; all the scales demonstrated good reliability and internal consistency.

An online survey was performed to facilitate participants’ comfort level, enabling them to express their opinions openly. An information document for the participants was included in the invitation email, which contained the survey link and outlined the participants’ rights. Furthermore, the email was distributed to the directors of the nursing departments at the selected hospitals, who subsequently forwarded the email to a total of 693 registered nurses and managers at their hospitals. Over a four-week data collection period, 511 nurses participated in survey, reflecting a response rate of 73.7%.

To reduce self-report and social desirability bias, the survey was administered anonymously through an online platform, and participants were informed that their responses would remain confidential and would not be shared with hospital management. Participation was voluntary, and the invitation emphasized that nurses could decline without any consequence. To further reduce response-pattern bias, the questionnaire items were presented in a standardized order, and participants completed the survey independently.

### 2.4. Data Analysis

All returned questionnaires were screened for completeness before analysis, and records with substantial missing data (N = 24) were excluded from the final dataset of 487 participants; only complete cases were included in the statistical analysis. SPSS Statistics for Windows version 24 (IBM Corp., Armonk, NY, USA) was used for data analysis. Descriptive statistics were used to outline the participants’ sociodemographic profile, analyze their levels of trust in nursing managers, and clarify the effect of their manager’s inclusive leadership style on their work effectiveness. Normality and correlation analysis. Prior to correlation analysis, the distribution of the composite scale scores was examined using Shapiro–Wilk tests, as well as inspection of skewness and kurtosis values. Although the composite scores showed statistically significant departures from normality, the distributions were reasonably symmetric and the summed scale scores were treated as approximately continuous for parametric analysis. Specifically, the Shapiro–Wilk values indicated non-normality across the composite variables, with skewness values ranging from −0.3225 to −0.0267 and kurtosis values ranging from −1.1654 to −0.7992. Therefore, Pearson’s correlation coefficient was used to examine the relationships among the study variables. As a sensitivity consideration, nonparametric correlations may also be reported where appropriate. Accordingly, Pearson’s correlation coefficient was used to examine the relationships between perceived trust in nurse managers, work effectiveness, and inclusive leadership styles. Independent samples *t*-tests were conducted to compare nursing managers and staff nurses on the study subscales using a two-tailed significance level of 0.05.

## 3. Results

### 3.1. Sociodemographic

Table 2 presents the sociodemographic characteristics of the participants, indicating that a significant portion of the participants were women (70%); most of the participants were married (73.5%). Most of the participants (65.3%) were 20–29 years old, and 29.8% were aged between 30 and 39. In terms of nationality, 17.7% were Saudi nationals, whereas Indians, Egyptians, and Filipinos were the most prominent among the rest. In addition, 46.6% of the participants held a diploma, whereas 28.7% had a bachelor’s degree. Among the participants, 77% were staff nurses and 23% were nursing managers. In addition, 47.8% of the participants had >7 years of work experience. A majority of the participants were from the eastern region (47%) of the country, and the rest were evenly distributed across other regions. Approximately 68.9% of the participants worked in public hospitals, and approximately 74.4% and 25.6% worked in 8 and 10–12 h shifts.

### 3.2. Perceptions of Work Effectiveness

The findings related to work effectiveness (Table 3) indicate that the dimensions of opportunity, access to support, and access to information (mean: 3.43, 3.23, and 3.15, respectively) were moderately empowered. The dimensions of access to resources, formal power, and global empowerment were relatively less empowered. In terms of work effectiveness, nursing managers perceived more empowerment across all dimensions compared with staff nurses (*p* < 0.05), except for access to support.

### 3.3. Perceptions of Inclusive Leadership Dimensions

In relation to an inclusive leadership style (Table 4), the overall scores indicated that while availability (mean: 3.43) and accessibility (mean: 3.23) were perceived by the majority of the participants, openness (mean: 2.81) was poorly or not effectively experienced. Moreover, a significant difference was observed between participants’ perceptions of all dimensions of inclusive leadership (*p* < 0.05), especially openness (*p* < 0.0001; *p* < 0.05).

### 3.4. Perceptions of Trust in Leaders

With regard to trust in leaders (Table 5), integrity (mean: 3.31) and benevolence (mean: 3.27) were more highly rated than predictability (mean: 2.79) and competence (mean: 2.81). Significant differences (*p* < 0.05) were observed between nursing managers (mean range: 3.26–3.57) and nurses (mean range: 2.64–3.19) across all dimensions of trust in leadership, where nursing managers perceived more trust in leadership than the nurses.

Independent samples *t*-tests showed that nursing managers reported significantly higher mean scores than staff nurses across all work effectiveness, inclusive leadership, and trust subscales, including opportunity, access to information, access to support, access accessibility, benevolence, integrity, predictability, and competence (all *p* < 0.01).

### 3.5. Correlations Between Trust in Leaders, CWES and Inclusive Leadership Subscales

Table 6 highlights the correlations between trust in leaders (benevolence, integrity, predictability, and competence) and all CWEQ subscales. Benevolence exhibited strong positive correlations with opportunity (r = 0.885, *p* < 0.01); access to information (r = 0.929, *p* < 0.01); access to support (r = 0.961, *p* < 0.01); and accessibility (r = 0.962, *p* < 0.01), suggesting a pivotal role in fostering empowerment and inclusivity. Meanwhile, integrity showed significant associations with opportunity (r = 0.962, *p* < 0.01), access to information (r = 0.869, *p* < 0.01), and availability (r = 0.952, *p* < 0.01), indicating an influence on resource access and leader or manager availability. Predictability was strongly correlated with formal power (r = 0.861, *p* < 0.01) and openness (r = 0.908, *p* < 0.01), highlighting its connection to structured leadership and open communication. Finally, competence was significantly correlated with access to information (r = 0.892, *p* < 0.01), access to resources (r = 0.746, *p* < 0.01), and global empowerment (r = 0.924, *p* < 0.01), reflecting its importance in resource allocation and empowerment. The subscales of openness, availability, and accessibility showed notable associations with multiple trust dimensions, particularly competence and predictability, emphasizing the role of accessible and open-minded leaders in promoting trust and empowerment. However, informal power showed weaker correlations across the trust dimensions, suggesting a limited influence in this context.

### 3.6. Correlations Between the CWES and Inclusive Leadership Subscales

The correlation between work effectiveness and inclusive leadership subscales (Table 7) reflected a strong positive relationship (r > 0.6, *p* < 0.01). A significant relationship was noted between access to support and availability (r = 0.856, *p* < 0.01); access to information and accessibility (r = 0.931, *p* < 0.01); formal power and openness (r = 0.882, *p* < 0.01); and opportunity and accessibility (r = 0.856, *p* < 0.01).

## 4. Discussion

This study examined the associations between staff nurses’ perceptions of trust in their managers, inclusive leadership styles, and work effectiveness in Saudi Arabian hospitals. The findings offer meaningful insights into how relational and leadership dynamics can shape staff empowerment and performance. While the study employed a cross-sectional correlational design—limiting the ability to infer causation—it nonetheless reveals significant associations that are consistent with prior theoretical and empirical literature.

The results showed that trust dimensions such as benevolence and integrity were rated higher by nurses than predictability and competence. These findings align with previous studies highlighting the emotional foundations of trust in leadership. Leaders perceived as honest and caring foster stronger relationships with staff, encouraging engagement and open communication [4,5,10]. In contrast, lower ratings in predictability and competence suggest that staff may be uncertain about their leaders’ consistency and technical capacity, echoing earlier findings that competency is a critical dimension in trust-building and performance outcomes [42,43,44,45].

Similarly, inclusive leadership dimensions such as availability and accessibility were perceived positively, while openness scored lower. This may reflect the cultural and professional complexities within the nursing workforce in Saudi Arabia, which includes diverse nationalities and professional backgrounds [42,43]. As Ashikali et al. [17] and Fang et al. [18] noted, inclusive leadership thrives when leaders actively embrace diversity and demonstrate consistent openness to staff input. Lower perceived openness could point to unaddressed communication barriers or perceived hierarchical distance between staff nurses and their managers.

The study also revealed that trust in leaders—particularly integrity and benevolence—was strongly associated with access to support, information, and opportunity, key subscales of structural empowerment. These associations echo research suggesting that when nurse leaders act with fairness and care, staff are more likely to perceive themselves as empowered [10,22,46]. For example, the link between benevolence and access to support is supported by findings from Firmansyah et al. [41], who emphasized that supportive leadership cultivates confidence in the workplace.

The strong correlations between inclusive leadership subscales (openness, availability, accessibility) and work effectiveness variables are also consistent with previous literature [47,48,49,50,51]. Inclusive leadership has been found to reduce psychological stress [47], improve innovation [50], and promote staff autonomy and engagement [26,27]. The significant relationships found in this study suggest that staff who perceive their leaders as accessible and inclusive are more likely to report higher levels of empowerment and effectiveness.

However, the relatively weaker association between trust dimensions like predictability and formal power or informal power suggests that staff may not fully perceive consistency or clarity in leadership decisions or relational influence. This is consistent with earlier studies emphasizing the importance of visible, consistent leadership behavior in fostering effective nurse management [52,53].

Several correlations in the present study were very high, particularly among trust, inclusive leadership, and structural empowerment subscales. Although these associations are statistically significant, coefficients above 0.90 suggest that some constructs may overlap conceptually or may have been influenced by shared method variance, halo effects, or other response biases inherent in self-report data. Accordingly, these findings should not be interpreted as evidence of clearly separable constructs in all cases. Instead, they indicate that the measured dimensions are closely related and may reflect a broader underlying leadership–empowerment experience in the nursing context.

Taken together, the findings indicate that while trust and inclusive leadership are clearly associated with key aspects of nurses’ work effectiveness, there are areas—such as predictability, openness, and informal power—where improvements could strengthen these associations. These results underscore the importance of cultivating inclusive, competent, and transparent leadership styles to improve nursing performance and organizational outcomes [19,28,54,55,56,57,58,59]. This study extends the limited Saudi nursing leadership literature by showing that trust, inclusive leadership, and work effectiveness are closely related in a multicultural hospital workforce. Compared with earlier Saudi studies that mainly described leadership values or focused on job satisfaction and safety culture, the present findings add evidence that relational leadership factors are also associated with empowerment-related outcomes in Saudi hospitals.

### 4.1. Study Limitations and Future Research

The study relied on self-reported data collected at a single time point, which increases the likelihood of common method bias and may partly explain the very high correlations observed among some subscales. The very high correlations observed among some subscales suggest substantial conceptual overlap between certain dimensions of trust, inclusive leadership, and structural empowerment. Although these measures were drawn from established instruments, formal discriminant validity testing was not conducted in the present study. Therefore, the findings should be interpreted cautiously, as some of the observed associations may reflect partial construct redundancy rather than wholly independent effects. Because the study examined several related subscales across three instruments, multiple inferential tests were conducted. These analyses were intended to be exploratory and theory-informed rather than confirmatory hypothesis tests. Therefore, findings are interpreted cautiously, with emphasis on effect sizes and pattern consistency rather than *p*-values alone. Future research should consider multiplicity-adjusted testing procedures or structural equation modeling to more rigorously evaluate the interrelationships among trust, inclusive leadership, and work effectiveness.

### 4.2. Theoretical and Practical Implications

The findings have both theoretical and practical implications for nursing leadership research and management practice. From a theoretical perspective, the study supports the relevance of Social Exchange Theory in explaining how trust-based and inclusive leader–staff relationships are associated with perceptions of work effectiveness in hospital settings. At the same time, the very high correlations observed among some subscales suggest that these constructs may not be fully distinct and may partly reflect conceptual overlap or shared method variance, indicating the need for future studies to test discriminant validity more rigorously. From a practical perspective, the findings are especially relevant to Saudi hospitals, where the nursing workforce is culturally and linguistically diverse. In such settings, inclusive leadership should be culturally responsive and supported by clear communication, equitable access to information and support, psychologically safe work environments, and leadership practices grounded in cultural humility and respect for workforce diversity. Together, these findings suggest that strengthening trust and inclusive leadership may help improve nurses’ workplace experiences, while future research should further refine the conceptual boundaries among these related constructs.

## 5. Conclusions

This study found that trust in nurse managers, inclusive leadership, and staff nurses’ work effectiveness were positively associated in Saudi Arabian hospitals. Staff nurses who perceived higher levels of trust in their managers also tended to report more favorable perceptions of inclusive leadership and work effectiveness. Likewise, inclusive leadership was associated with stronger perceptions of work effectiveness, suggesting that relational and supportive leadership practices may be important within this workforce. These findings provide useful evidence for hospital administrators and nursing leaders seeking to strengthen workplace relationships and staff outcomes in multicultural hospital settings.

However, because the study used a descriptive cross-sectional design, the results should be interpreted as associations rather than causal effects. Future research using longitudinal or intervention-based designs is needed to examine how these variables influence one another over time and to test whether inclusive leadership outcomes change when trust-based leadership practices are strengthened. In addition, further studies should explore potential contextual and cultural factors that may shape these relationships in Saudi hospitals.

## Figures and Tables

**Table 1:** Reliability of the questionnaires.

Questionnaire	Subscales	Cronbach’s Alpha
Workplace effectiveness questionnaire II	Opportunity	0.82
	Access to information	0.93
	Access to support	0.91
	Access to resources	0.98
	Formal power	0.94
	Informal power	0.86
	Global empowerment	0.94
Inclusive leadership	Openness	0.97
	Availability	0.95
	Accessibility	0.83
Trust in the leader (manager)	Benevolence	0.88
	Integrity	0.96
	Predictability	0.96
	Competence	0.82

**Table 2:** Sociodemographic data of the study participants.

Items	Groups	Nurse Managers (*n* = 112)		Staff Nurses (*n* = 375)		Total (*n* = 487)	
		*n*	%	*n*	%	*n*	%
Age (in years)	20–29	12	10.7%	245	65.3%	257	52.8%
	30–39	39	34.8%	106	28.3%	145	29.8%
	40–49	57	50.9%	19	5.1%	76	15.6%
	50–59	4	3.6%	5	1.3%	9	1.8%
Gender	Male	23	20.5%	123	32.8%	146	30%
	Female	89	79.5%	252	67.2%	341	70%
Marital status	Single	12	10.7%	101	26.9%	122	25.1%
	Married	94	83.9%	264	70.4%	358	73.5%
	Widowed/divorced	6	5.4%	10	2.7%	16	3.4%
Nationality	Saudi	45	40.2%	41	10.9%	86	17.7%
	Indian	39	34.8%	64	17.1%	103	21.1%
	Egyptian	13	11.6%	92	24.5%	105	21.6%
	Filipino	9	8.0%	78	20.8%	87	17.9%
	Sudanese	2	1.8%	55	14.7%	57	11.7%
	Others	4	3.6%	45	12.0%	49	10.1%
Education	Diploma	17	15.2%	210	56.0%	227	46.6%
	Bachelor’s degree	59	52.7%	81	21.6%	140	28.7%
	Master’s degree	32	28.6%	60	16.0%	92	18.9%
	Doctoral degree	4	3.6%	2	0.5%	6	1.2%
	Others	0	0.0%	22	5.9%	22	4.5%
Work experience	1–2	0	0.0%	53	14.1%	53	10.9%
	3–6	2	1.8%	199	53.1%	201	41.3%
	7–10	84	75.0%	48	12.8%	132	27.1%
	>10	26	23.2%	75	20.0%	101	20.7%
Region	Central	12	10.7%	50	13.3%	62	12.7%
	Northern	24	21.4%	48	12.8%	72	14.8%
	Eastern	47	42.0%	182	48.5%	229	47.0%
	Southern	19	17.0%	34	9.1%	53	10.9%
	Western	10	8.9%	61	16.3%	71	14.6%

**Table 3:** Perceptions of work effectiveness (*n* = 487).

Subscale	Total Mean	Nurse Managers		Nurses		t-Value	*p*-Value	Cohen’s d
		Mean	SD	Mean	SD			
Opportunity	3.43	3.74	1.00	3.34	1.29	3.5146	0.0005 *	0.33
Access to information	3.15	3.56	0.98	3.03	1.28	4.6342	<0.0001 *	0.43
Access to support	3.23	3.49	1.04	3.16	1.29	2.7247	0.007 *	0.26
Access to resources	2.75	3.15	0.91	2.63	1.03	5.1124	<0.0001 *	0.52
Formal power	2.75	3.17	1.09	2.63	1.00	4.665	<0.0001 *	0.53
Informal power	3.07	3.37	0.88	2.99	0.82	4.1067	<0.0001 *	0.46
Global empowerment	2.79	3.21	1.18	2.67	1.03	4.3885	<0.0001 *	0.51

* Significant difference.

**Table 4:** Perceptions of inclusive leadership dimensions (*n* = 487).

Dimensions	Total Mean	Nurse Managers		Nurses		t-Value	*p*-Value	Cohen’s d
		Mean	SD	Mean	SD			
Openness	2.81	3.29	1.09	2.66	1.01	5.4659	<0.0001 *	0.61
Availability	3.43	3.74	1.00	3.34	1.29	3.5146	0.0005 *	0.33
Accessibility	3.23	3.49	1.04	3.16	1.29	2.7247	0.007 *	0.26

* Significant difference.

**Table 5:** Perceptions of trust in leaders (*n* = 487).

	Total Mean	Nurse Managers		Nurses		t-Value	*p*-Value	Cohen’s d
		**Mean**	SD	Mean	SD			
Benevolence	3.27	3.57	0.99	3.18	1.22	3.48	0.0006 *	0.34
Integrity	3.31	3.66	0.93	3.20	1.22	4.2683	<0.0001 *	0.4
Predictability	2.79	3.28	1.00	2.64	0.99	5.9613	<0.0001 *	0.65
Competence	2.81	3.26	0.98	2.67	0.99	5.6237	<0.0001 *	0.6

* Significant difference.

**Table 6:** Correlations between trust in leaders, CWES and inclusive leadership subscales.

Subscale	Benevolence	Integrity	Predictability	Competence
Opportunity		0.962 **	0.135	0.107
Access to information	0.929 **	0.869 **	0.703 **	0.892 **
Access to support	0.961 **	0.887 **	0.097	0.084
Access to resources	0.921 **	0.086	0.136	0.746 **
Formal power	0.093	0.121	0.861 **	0.869 **
Informal power	0.099	0.077	0.103	0.084
Global empowerment	0.084	0.684 **	0.835 **	0.924 **
Openness	0.614 **	0.108	0.908 **	0.965 **
Availability	0.887 **	0.952 **	0.135	0.724 **
Accessibility	0.962 **	0.837 **	0.098	0.782 **

** Correlation is significant at the 0.01 level (two-tailed).

**Table 7:** Correlations between the CWES and inclusive leadership subscales.

Subscale	Openness	Availability	Accessibility
Opportunity	0.609 **	0.748 **	0.856 **
Access to information	0.691 **	0.777 **	0.931 **
Access to support	0.682 **	0.856 **	0.721 **
Access to resources	0.748 **	0.681 **	0.673 **
Formal power	0.882 **	0.712 **	0.776 **
Informal power	0.623 **	0.601 **	0.784 **
Global empowerment	0.794 **	0.692 **	0.681 **

** Correlation is significant at the 0.01 level (two-tailed).

## Data Availability

The data sets used and/or analyzed during the current study are presented in the results and manuscript. Further reasonable requests should be directed to the corresponding author.

## References

[1] Molina-Mula J., Gallo-Estrada J. (2020). Impact of nurse-patient relationship on the quality of care and patient autonomy in decision-making. Int. J. Environ. Res. Public Health.

[2] Corbaz-Kurth S., Weissbrodt R., Juvet T.M., Hannart S., Krsmanovic B., Plaschy I.S., Terrier P. (2025). How does trust emerge in interprofessional collaboration? A qualitative study of the significance, importance, and dynamics of trust in healthcare teams and networks. J. Interprof. Care.

[3] Sutherland B.L., Pecanac K., LaBorde T.M., Bartels C.M., Brennan M.B. (2021). Good working relationships: How healthcare system proximity influences trust between healthcare workers. J. Interprof. Care.

[4] Zak P. (2019). The neuroscience of trust. Harv. Bus. Rev..

[5] Eliyana A., Ma’arif S., Muzakki (2019). Job satisfaction and organizational commitment effect in the transformational leadership toward employee performance. Eur. Res. Manag. Bus. Econ..

[6] Håvold O.K.S., Håvold J.I., Glavee-Geo R. (2020). Trust in leaders, work satisfaction and work engagement in public hospitals. Int. J. Public Leadersh..

[7] Hadi-Moghaddam M., Karimollahi M., Aghamohammadi M. (2021). Nurses’ trust in managers and its relationship with nurses’ performance behaviors: A descriptive-correlational study. BMC Nurs..

[8] González-Navarro P., Zurriaga-Llorens R., Tosin Olateju A., Llinares-Insa L.I. (2018). Envy and counterproductive work behavior: The moderation role of leadership in public and private organizations. Int. J. Environ. Res. Public Health.

[9] Thazha S.K., Cruz J.P., Alquwez N., Scaria B., Rengan S.S., Almazan J.U. (2022). Infection prevention and control awareness, attitudes, and practices among healthcare professionals in South India. J. Infect. Dev. Ctries..

[10] Nurmeksela A., Mikkonen S., Kinnunen J., Kvist T. (2021). Relationships between nurse managers’ work activities, nurses’ job satisfaction, patient satisfaction, and medication errors at the unit level: A correlational study. BMC Health Serv. Res..

[11] Farmanesh P., Zargar P., Levine M.P. (2021). Trust in leaders as a psychological factor on employee and organizational outcome. The Psychology of Trust.

[12] Alsadaan N., Salameh B., Reshia F.A.A.E., Alruwaili R., Alruwaili M., Awad Ali S., Alruwaili A., Hefnawy G., Alshammari M., Alruwaili A. (2023). Impact of nurse leaders behaviors on nursing staff performance: A systematic review of literature. Inquiry.

[13] Flores I.F., Dator W.L.T., Olivar J.J., Gaballah M.K. (2023). Congruence of effective leadership values between nurse leaders and staff nurses in a multicultural medical city in Saudi Arabia: A sequential mixed-methods study. Healthcare.

[14] Veli Korkmaz A., Van Engen M.L., Knappert L., Schalk R. (2022). About and beyond leading uniqueness and belongingness: A systematic review of inclusive leadership research. Hum. Resour. Manag. Rev..

[15] Ammar H.E.S., Keshk L.I.Z., Dahshan M.E.A.E. (2025). Inclusive leadership and its relation to teamwork practices and patients’ safety culture from nurses’ perspective. Deleted J..

[16] Fu Q., Cherian J., Ahmad N., Scholz M., Samad S., Comite U. (2022). An inclusive leadership framework to foster employee creativity in the healthcare sector: The role of psychological safety and polychronicity. Int. J. Environ. Res. Public Health.

[17] Ashikali T., Groeneveld S., Kuipers B. (2020). The role of inclusive leadership in supporting an inclusive climate in diverse public sector teams. Rev. Public Pers. Adm..

[18] Fang Y.C., Chen J.Y., Wang M.J., Chen C.Y. (2019). The impact of inclusive leadership on employees’ innovative behaviors: The mediation of psychological capital. Front. Psychol..

[19] Nikpour J., Hickman R.L., Clayton-Jones D., Gonzalez-Guarda R., Broome M.E. (2022). Inclusive leadership to guide nursing’s response to improving health equity. Nurs. Outlook.

[20] Jankelová N., Joniaková Z. (2021). Communication skills and transformational leadership style of first-line nurse managers in relation to the job satisfaction of nurses and moderators of this relationship. Healthcare.

[21] Rosen M.A., DiazGranados D., Dietz A.S., Benishek L.E., Thompson D., Pronovost P.J., Weaver S.J. (2018). Teamwork in healthcare: Key discoveries enabling safer, high-quality care. Am. Psychol..

[22] Burke C.S., Sims D.E., Lazzara E.H., Salas E. (2007). Trust in leadership: A multi-level review and integration. Leadersh. Q..

[23] Mishra A.K., Mishra K.E. (2013). The research on trust in leadership: The need for context. J. Trust. Res..

[24] Legood A., van der Werff L., Lee A., Den Hartog D. (2020). A meta-analysis of the role of trust in the leadership-performance relationship. Eur. J. Work. Organ. Psychol..

[25] Alrasheadi B.A., Alamri M.S., Aljohani K.A., Al-Dossary R., Albaqawi H., Alharbi J., Hosis K.A., Aljohani M.S., Almadani N., Falatah R. (2022). Nurses’ perception of safety culture in medical-surgical units in hospitals in Saudi Arabia. Medicina.

[26] Adolfo C., Albougami A., Roque M., Almazan J. (2021). Nurses’ attitudes toward quality improvement in hospitals: Implications for nursing management systems. Nurs. Pract. Today.

[27] Aljohani K.A., Alamri M.S., Al-Dossary R., Albaqawi H., Hosis K.A., Aljohani M.S., Almadani N., Alrasheadi B., Falatah R., Almazan J. (2022). Scope of nursing practice as perceived by nurses working in Saudi Arabia. Int. J. Environ. Res. Public Health.

[28] Specchia M.L., Cozzolino M.R., Carini E., Di Pilla A., Galletti C., Ricciardi W., Damiani G. (2021). Leadership styles and nurses’ job satisfaction: Results of a systematic review. Int. J. Environ. Res. Public Health.

[29] Lee S.E., Seo J. (2024). Effects of nurse managers’ inclusive leadership on nurses’ psychological safety and innovative work behavior: The moderating role of collectivism. J. Nurs. Scholarsh..

[30] Huang H., Zhang X., Tu L., Peng W., Wang D., Chong H., Wang Z., Du H., Chen H. (2024). Inclusive leadership, self-efficacy, organization-based self-esteem, and intensive care nurses’ job performance: A cross-sectional study using structural equation modeling. Intensive Crit. Care Nurs..

[31] Alilyyani B. (2022). The effect of authentic leadership on nurses’ trust in managers and job performance: A cross-sectional study. Nurs. Rep..

[32] Shore L.M., Chung B.G. (2022). Inclusive leadership: How leaders sustain or discourage work group inclusion. Group Organ. Manag..

[33] Abualruz H., El-Gazar H., Al-Ghabeesh S., Tabar N.A., Alshariah H., Abousoliman A. (2023). The impact of utilizing inclusive leadership on nurses during crises: A multisite comparative study. J. Med. Life.

[34] Grimani A., Aboagye E., Kwak L. (2019). The effectiveness of workplace nutrition and physical activity interventions in improving productivity, work performance and workability: A systematic review. BMC Public Health.

[35] Aburumman M., Newnam S., Fildes B. (2019). Evaluating the effectiveness of workplace interventions in improving safety culture: A systematic review. Saf. Sci..

[36] Alsadaan N., Jones L.K., Kimpton A., DaCosta C. (2021). Challenges Facing the Nursing Profession in Saudi Arabia: An Integrative Review. Nurs. Rep..

[37] Davies J., Yarrow E., Callaghan S. (2025). A narrative review of nursing in Saudi Arabia: Prospects for improving social determinants of health for the female workforce. Front. Public Health.

[38] Xerri M. (2012). Workplace relationships and the innovative behaviour of nursing employees: A social exchange perspective. Asia Pac. J. Hum. Resour..

[39] Madison K., Eva N., De Cieri H., Goh Z. (2025). Social exchange theory in leadership research: A problematizing review. Leadersh. Q..

[40] Titan I. (2016). Comparison of convenience sampling and purposive sampling. Am. J. Theor. Appl. Stat..

[41] Woolson R.F., Bean J.A., Rojas P.B. (1986). Sample size for case-control studies using Cochran’s statistic. Biometrics.

[42] Laschinger H. (2012). Conditions for Work Effectiveness Questionnaire I and II: User Manual.

[43] Ramos O.A.M., De Sá J.A.F.T.M., Gomes J.M.P.A., Galvão A.M.N.P., Gomes M.J.A.R. (2025). Influence of leadership style on nurses’ structural empowerment: A cross-sectional study. Cogitare Enferm..

[44] Carmeli A., Reiter-Palmon R., Ziv E. (2010). Inclusive leadership and employee involvement in creative tasks in the workplace: The mediating role of psychological safety. Creat. Res. J..

[45] Firmansyah M.R., Amelia R., Jamil R.A., Faturochman F., Minza W.M. (2019). Which one is more influential on trust in friendships?. J. Psikol..

[46] Ta’an W.F., Al-Hammouri M.M., Rababah J.A., Suliman M.M. (2021). Reliability and validation of the Arabic version of the Conditions for Workplace Effectiveness Questionnaire-II. Int. J. Nurs. Sci..

[47] Al-Atwi A.A., Al-Hassani K.K. (2021). Inclusive leadership: Scale validation and potential consequences. Leadersh. Organ. Dev. J..

[48] Kattan W., Al-Hanawi M.K. (2025). Inequalities in the distribution of the nursing workforce in the Kingdom of Saudi Arabia: A regional analysis. Hum. Resour. Health.

[49] Halabi J.O., Lepp M., Nilsson J. (2020). Assessing self-reported competence among registered nurses working as a culturally diverse workforce in public hospitals in the Kingdom of Saudi Arabia. J. Transcult. Nurs..

[50] Carter B. (2020). Achieving diversity, inclusion and equity in the nursing workforce. Rev. Lat. Am. Enferm..

[51] Phillips J.M., Malone B. (2014). Increasing racial/ethnic diversity in nursing to reduce health disparities and achieve health equity. Public Health Rep..

[52] Freire C., Azevedo R. (2015). Empowering and trustful leadership: Impact on nurses’ commitment. Pers. Rev..

[53] Ahmed F., Zhao F., Faraz N.A. (2020). How and when does inclusive leadership curb psychological distress during a crisis? Evidence from the COVID-19 outbreak. Front. Psychol..

[54] Ahmed F., Zhao F., Faraz N.A., Qin Y.J. (2021). How inclusive leadership paves way for psychological well-being of employees during trauma and crisis: A three-wave longitudinal mediation study. J. Adv. Nurs..

[55] Zeng D., Wang B., Chen W. (2023). Inclusive leadership can improve nurses’ psychological ownership and reduce their turnover intention under the normalization of COVID-19 prevention. Front. Psychol..

[56] Elsayed W., Abdel-Ghani A., Ibrahim A. (2020). The role of work locus of control and inclusive leadership on nurses’ innovative work behavior. Assiut. Sci. Nurs. J..

[57] Henshall C., Jones L., Armitage C., Tomlinson L. (2022). Empowering nurses through inclusive leadership to promote research capacity building: A James Lind Alliance priority setting partnership in community nursing. J. Adv. Nurs..

[58] Prestia A.S. (2020). Contemplating the importance of trust. Nurse Lead..

[59] Sherman R.O. (2023). Rebuilding trust in nursing leadership. Nurse Lead..

